# Characterization of a Missense Mutation in the Catalytic Domain and a Splicing Mutation of Coagulation Factor X Compound Heterozygous in a Chinese Pedigree

**DOI:** 10.3390/genes12101521

**Published:** 2021-09-27

**Authors:** Yuanzheng Feng, Jiewen Ma, Liang V Tang, Wenyi Lin, Yanyi Tao, Zhipeng Cheng, Yu Hu

**Affiliations:** Institute of Hematology, Wuhan Union Hospital, Tongji Medical College, Huazhong University of Science and Technology, No. 1277 Jiefang Avenue, Wuhan 430022, China; hustfyz2019@hust.edu.cn (Y.F.); jiewen_ma@hust.edu.cn (J.M.); dr_tang2020@163.com (L.V.T.); wenyilin0221@163.com (W.L.); yanyit@hust.edu.cn (Y.T.); czp_325@163.com (Z.C.)

**Keywords:** factor X, deficiency, compound heterozygous mutations, coagulation, bleeding

## Abstract

Background: Congenital coagulation factor X (FX) deficiency is a rare bleeding disorder with an incidence of one in one million caused by mutations in the FX-coding gene(F10), leading to abnormal coagulation activity and a tendency for severe hemorrhage. Therefore, identifying mutations in FX is important for diagnosing congenital FX deficiency. Results: Genetic analysis of the proband identified two single-base substitutions: c.794T > C: p.Ile265Thr and c.865 + 5G > A: IVS7 + 5G > A. His FX activity and antigen levels were < 1% and 49.7%, respectively; aPTT and PT were prolonged to 65.3 and 80.5 s, respectively. Bioinformatics analysis predicted the two novel variants to be pathogenic. In-vitro expression study of the missense mutation c.794T > C: p.Ile265Thr showed normal synthesis and secretion. Activation of FXs by RVV, FVII/TF, and FVIII/FIX all showed no obvious difference between the variant and the reference. However, clotting activity by PT and aPTT assays and activity of thrombin generation in a TGA assay all indicated reduced activity of the mutant FX-Ile265Thr compared to FX-WT. Minigene assay showed a normal splicing mode c.865 + 5G > A: IVS7 + 5G > A, which is inconsistent with clinical phenotype. Conclusions: The heterozygous variants c.794T > C: p.Ile265Thr or c.865 + 5G > A: IVS7 + 5G > A indicate mild FX deficiency, but the compound heterozygous mutation of the two causes severe congenital FX deficiency. Genetic analysis of these two mutations may help characterize the bleeding tendency and confirm congenital FX deficiency. In-vitro expression and functional study showed that the low activity of the mutant FX-Ile265Thr is caused by decrease in its enzyme activity rather than self-activation. The minigene assay help us explore possible mechanisms of the splicing mutation. However, more in-depth mechanism research is needed in the future.

## 1. Introduction

The coagulation Factor X (FX) is a vitamin K-dependent zymogen of serine protease synthesized by the liver. It is a disulfide-bonded two-chain glycoprotein consisting of a 17-kDa light chain and a 45-kDa heavy chain [1]. In the coagulation cascade, both the intrinsic (FIXa/FVIIIa) and the extrinsic pathway (FVIIa/tissue factor) activate FX to activated FX (FXa) [2,3]. Prothrombin is then converted to thrombin by the assemble of FXa, activated factor V (FVa) and Ca^2+^ to form prothrombinase complex, which is the only physiological activator of prothrombin in vivo [4]. FX deficiency is one of the most severe coagulation factor deficiencies, second only to that of hemophilia A and hemophilia B. Most mice with homozygous knockout of the F10 die from fatal bleeding during the embryonic and neonatal stages, and the remaining will not survive till weaning [5]. While heterozygotes are often asymptomatic, homozygotes and compound heterozygotes are prone to severe bleeding, depending on the degree of reduced FX activity [6,7,8,9].

Congenital FX deficiency is autosomal recessive, and the incidence is about one in million in general population [10,11,12]. Most recent data derived from the World Federation of Hemophilia (WFH) global survey and the Rare Bleeding Disorders Database survey (RBDD) showed that patients with FX deficiency represent 8% of the total number of patients suffering from rare bleeding disorders worldwide (2497 vs. 30,166). Since the first two cases of FX deficiency (Stuart and Prower) caused by congenital variants were reported, 176 different types of genetic mutations in F10 had been reported around the world according to the Human Gene Mutation Database (HGMD) professional 2020.3 (https://portal.biobase-international.com (accessed on 30 April 2021). Among all the types of genetic mutation, missense and nonsense mutations caused by single-base substitutions were the most prevalent, accounting for 78% (130 cases) of all mutations. Only a few variants have been reported in the Chinese population, wherein there may be many unreported variants that deserve notice [13,14,15,16,17,18,19,20,21,22,23]. In this study, we present a compound heterozygous congenital FX deficiency identified in a patient, along with our exploration of the functional defect caused by mutation.

## 2. Materials and Methods

### 2.1. Nomenclature

Variants found in the patient are reported according to the Human Genome Variation Society (HGVS) nomenclature, with nucleotide numbering of F10 sequence (reference NM_000504.3) starting at the ATG translation initiation codon. The A of the initiation codon ATG in the reference cDNA is numbered +1 and the Met in the corresponding protein reference is numbered +1. Reference transcript and protein are NM_000504.3 and NP_000495.1, respectively. The RefSeqGene is LRG_548.

### 2.2. Patients and Samples

The proband was a 28-year-old Chinese male admitted to hospital for tibia fracture. Plate fixation was successfully implemented, accompanied by an infusion of prothrombin complex concentrate (PCC) every day. He had a history of spontaneous gum bleeding and nose bleeding in childhood but never had major bleeding manifestation. He was diagnosed with FX deficiency at the age of 10 due to a hematoma in the right groin and started the administration of PCC irregularly. His parents were not consanguineous. No other family members have experienced bleeding symptoms. In total, we studied 10 family members of his. The venous blood samples from the proband and some of his family members were collected using vacutainer tubes (Zhiyuan, Wuhan, Hubei, China) containing citrate for all the tests. The sodium citrate samples were then centrifuged at 2200× *g* for 10 min to obtain the plasma and peripheral blood leukocytes.

### 2.3. Routine Coagulation and Coagulation Factor Assays

The plasma-activated partial thromboplastin time (aPTT), prothrombin time (PT), thromboplastin time (TT), and fibrinogen levels from the blood samples were measured using Stago STA-R Evolution (Asnières-Sur-Seine, France). The factor X coagulation activity (FX: C) assay method involved a one-stage prothrombin time (PT) analysis using thromboplastin and factor X-deficient reagents from Diagnostic Stago (Asnières-Sur-Seine, France). A mixing study was performed to eliminate the presence of inhibitors. FX concentrations were determined using enzyme-linked immunosorbent assay (ELISA, human coagulation factor X ELISA kit; ELK Biotechnology, Wuhan, China). Quality controls for all tests were performed according to the manufacturer’s instructions.

### 2.4. Gene Analysis

*DNA Extraction* Genomic DNA was extracted from the peripheral blood leukocytes of the proband and some of his family members using a DNA Blood Midi Kit (TsingKe, Beijing, China) and saliva collection bottle (Charmbiotech, Wuhan, China) was used for DNA collection and extraction of other family members.

*Sanger sequencing* Each exon, intron/exon boundary (~100 bps), and 3′-untranslated and 5′-flanking regions, including 500 bp nucleotides within the promoter was sequenced. PCR primers were constructed to amplify the promoter region and all eight exons including the exon-intron junction areas of the F10 (GenBank accession No. NG_009258.1). The PCR products were purified from agarose gel, and then sequenced using an ABI 3700 sequencer (Applied Biosystems, Foster City, CA, USA). All the PCR primers used for sequencing are listed in Supplementary Table S1.

### 2.5. Construction of Plasmid Expression Vectors

Modified F10 carrying the variant identified in the proband was generated. For this purpose, the full-length FX cDNA (1.5 kb) was first cloned into pcDNA3.1 (Thermo Fisher Scientific KK). Using this FX expression vector as a template, megaprimer PCR with mutagenic primers that contain base substitution was then carried out. The resulting vectors expressed the following proteins: wild type FX (FX-WT), FX with a substitution c.794T > C p.Ile265Thr (FX-Ile265Thr). The empty vector (pcDNA3.1) with no FX cDNA inserted was used as a mock control. The introduction of correct variant was verified by DNA sequencing.

### 2.6. Transfection

FX expression vectors were independently introduced into Hek293T cells using Lipofectamine3000 (Thermo Fisher Scientific KK). The vector expressing FX-WT and the mock vector were introduced into the cells and used as controls. After transfection and culture for 16 h, 3 times wash with phosphate-buffered saline (PBS) was applied and the medium was changed to serum-free DMEM for following culture for 24 h. Following this culture period, both cells and culture media were harvested. Cells were washed with PBS and homogenized in lysis buffer(Radio immunoprecipitation assay lysis buffer). After centrifugation of 12,000 rpm, 4 °C, for 15 min, supernatants were collected and used as cell lysate samples. Harvested culture medium were also centrifuged, and supernatants were used as assay samples. The collected samples were used to test the activity and concentrations of FX. SDS-PAGE analysis under non-reducing conditions was used to analyze FX in plasma of the proband and his parents, as well as in cells and culture medium. the concentrations of FX in cell lysates and culture medium were determined by Enzyme-linked immunosorbent assay.

### 2.7. Purification of Recombinant FX-WT and FX-Ile265Thr

The expression and purification of recombinant FX-WT and FX-Ile265Thr were conducted in human embryonic kidney (HEK-293) cells. The expression vectors were constructed as described above with 6 more His-tag at the C-terminal of the target protein. The recombinant FXs were isolated from 500 mL cell culture supernatants by immobilized metal affinity chromatography (Qiagen).

### 2.8. Activation of Recombinant FX

Recombinant FX-variant was activated by three different ways and compared directly with recombinant FX-WT in each case. Each FX (150 nM) in assay buffer (10 mM HEPES, pH 7.5, 100 mM NaCl, 5 mM CaCl2, 1 mg/mL bovine serum albumin, 1 mg/mL polyethyleneglycol 8000) was activated by (1) 100 nM RVV, Russell’s Viper Venom (Enzyme Research Laboratories, Indiana, USA); (2) 60 pM FVIIa(Novo Nordisk, Bagsværd, Denmark) in the presence of 500 pM lipidated recombinant tissue factor(Bio-Techne, Minneapolis, MN, USA) and (3) 5 nM FIXa(Enzyme Research Laboratories, Indiana, USA) in the presence of 4 units/mL of FVIIIa (Hualan Biological Engineering, Xinxiang, China) on 20 μM PE:PS:PC vesicles(5:3:2 *w*/*w*)(Avanti Polar Lipids, AL, USA). Activation of FX was monitored by quenching samples over time into assay buffer, with 5 mM EDTA and measuring the rate of hydrolysis of 100 mM S-2765(Hyphen biomed, Neuville sur Oise, France) in a multifunctional microplate reader PerkinElmer Enspire(PerkinElmer chemagen, Aachen, Germany)

### 2.9. Clotting Activity

The clotting activities of both recombinant wild-type and the recombinant FX variant (rFX) were evaluated by both PT and aPTT assays using a Stago STA-R Evolution (Diagnostica Stago, Asnieres, France) as described. The clotting activities of samples in both assays were assessed at five different dilutions ranging from 0.2 to 3.2μg/mL FX (final concentrations).

### 2.10. Thrombin Generation Assay

The thrombin generation activity of rFX variant and control was conducted in FX-deficient plasma following manufacturer’s protocol, reagents (Technoclone Herstellung von Diagnostika und Arzneimitteln GmbH, Vienna, Austria) and relative fluorescence units was read in a multifunctional microplate reader (PerkinElmer Enspire). The final concentration of FX in plasma was 8 μg/mL. Five parameters, including lag time, time to peak (TTP), peak height (Peak, nM), area under the curve, referred to the endogenous thrombin potential (ETP, nM*min) and the velocity index (VI), defined as VI = [peak height/(time to peak–lag time)] were used to assess thrombin generation dynamics using the ceveron α TGA software.

### 2.11. Minigene Assay

To study the splicing mutation IVS7 + 5G > A in FX, we used reverse transcription combined with nested PCR to analyze the patient’s periphery FX ectopic transcript. For the in-vitro splicing study, the minigene pcMINI-FX-WT/Mut was constructed, and modified pcDNA3.1 with double promoters (CMV promoter and T7 promoter) was used (Thermo Fisher Scientific KK) as vectors. The pcMINI-FX-WT/Mut contains exon7 and intron/exon boundaries, as well as exonA and exonB. ExonA and ExonB are sequences with strong splicing recognition used for splicing study before. We constructed two pairs of nested primers and use genomic DNA as the template for nested PCR. The nested PCR products of the second round were used as templates to amplify wild-type and mutant pcMINI fragments of 960 bp. Vector pcMINI and fragments were digested, recovered, ligated, and transformed into colonies for PCR identification and sequencing. 48 h after transfection of wild-type and mutant recombinant minigene into two commonly used mammalian cell lines for expression studies, MCF-7 and Hek-293T cells, the total RNA of the cells was extracted. After reverse transcription synthesis of cDNA, we used primers on both sides of minigene for PCR amplification. Agarose gel was used to detect the size of products. Sanger sequencing was also performed. All the PCR primers used are listed in Supplementary Table S1.

### 2.12. Bioinformatics Analysis

Varcards (http://varcards.biols.ac.cn/ (accessed on 30 April 2021) [24] was used To predict the pathogenicity of missense mutation c.794T > C in F10, which includes most commonly used predictive tools, such as SIFT, Polyphen-2, MutationTaster. NNSPLICE (https://www.frui-tfly.org/seq_tools/splice.html (accessed on 30 April 2021) and Netgene2 (http://www.cbs.dtu.dk/sevices/NetGene-2/output.php (accessed on 30 April 2021)) were used to predict the pathogenicity of splicing-site mutation c.865 + 5G > A. The species conservation of FX amino acid sequence is done in DNAMAN and presented with a picture in Pymol using the structure (PDB number 1c5m) in Protein Data Bank (http://www.rcsb.org (accessed on 30 April 2021).

## 3. Results

### 3.1. Genetic Analysis

**Figure 1:**
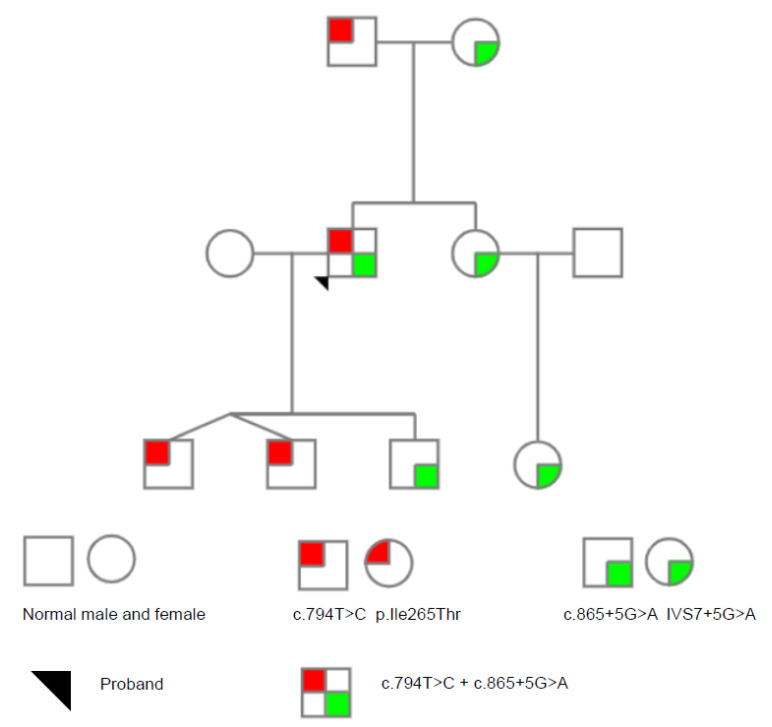
The pedigree of the proband with FX deficiency. The proband is compound heterozygous with c.794T > C and c.865 + 5G > A. Genetic analysis of the family members showed that his mother, sister, niece and elderly son are heterozygous for c.865 + 5G > A. His father and twin sons are heterozygous for c.865 + 5G > A.

**Figure 2:**
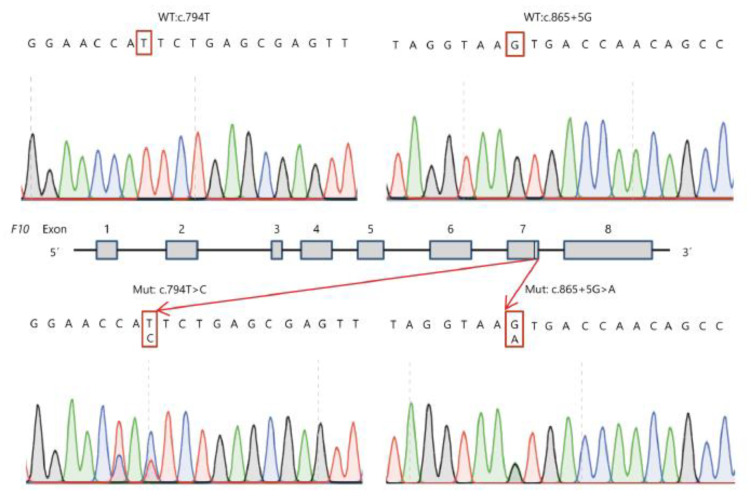
Sequencing analysis of the family. The proband has two types of mutation in exon7 and intron7: c.794T > C and c.865 + 5G > A. The analysis identified heterozygous mutations with c.794T > C in his father and twin sons and with c.865 + 5G > A in his mother, sister, niece and elderly son. WT = wild type, Mut = mutation.

Sanger Sequencing and family analysis showed that the proband carried compound heterozygous variants: FX:NG_009258.1:g.29627T > C (Genomic description)*;* NM_000504.3: c.794T > C(Transcript description); NP_000495.1:p.Ile265Thr(Protein description), a missense mutation, and FX:NM_000504.3 (c.865 + 5G > A: IVS7 + 5G > A), a splicing-site mutation. His father and elderly son carried the c.794T > C: p.Ile265Thr variant. His mother, sister, niece, and twin sons carried the c.865 + 5G > A: IVS7 + 5G > A variant. Figure 1 shows the family pedigree, and Figure 2 shows the sequencing analysis of the two variants found in the family. Besides, no other variants were found in F10 except a synonymous mutation c.792C > T.

### 3.2. Routine Coagulation Test and Coagulation Factors

The demographics and coagulation parameters of the proband and family members are shown in Table 1. The aPTT and PT of the proband were significantly prolonged to 65.3 and 80.5 s, respectively. For the heterozygous c.794T > C or c.865 + 5G > A alone aPTT and PT were in normal reference intervals. Other routine coagulation tests and coagulation factors of the proband, were all in normal ranges. The prolonged aPTT and PT of the proband were corrected by mixing studies, indicating that there were no coagulation factor inhibitors or lupus anticoagulants. The FX activity of the proband was severely reduced to less than 1% (reference interval: 70–120%). The FX activity of heterozygous c.794T > C was 57.2% (the proband’s father) and slightly less than 50% for c.865 + 5G > A (43.2%, 45.3%, and 42.3% respectively for the proband’s mother, sister, and elderly son). The test of FX concentration showed a similar trend as the FX activity, (see the detailed results in Table 1).

### 3.3. In-Vitro Expression

**Figure 3:**
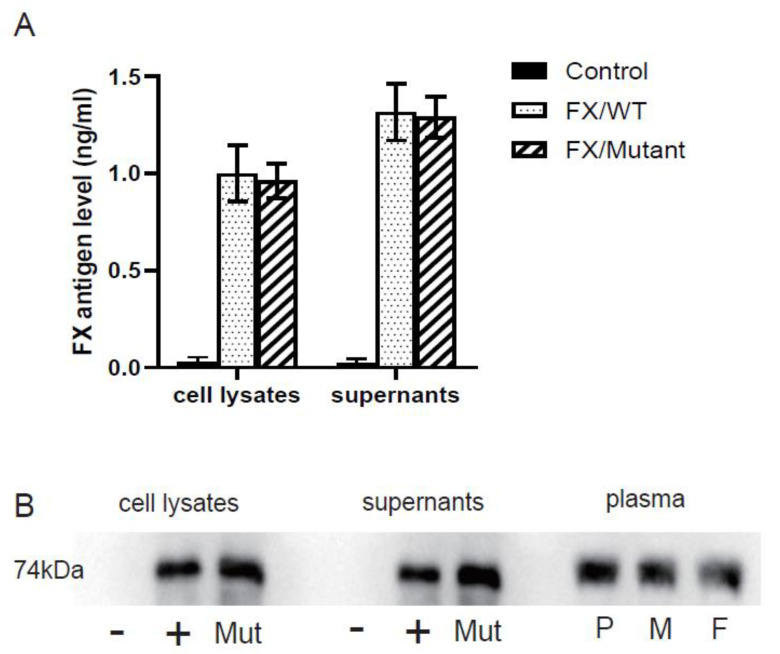
(**A**). FX antigen levels of the transfected cells in cell lysates and supernatants using ELISA. Data are derived from four independent measurements (SD). (**B**). Western Blot for the FX in transfected cell lysates and supernatants, as well in plasma of the proband and his parents. (-: control (empty vector); +: pcDNA3.1/FX WT; Mut: pcDNA3.1/FX Mut Thr265; P: Proband; M: Mother; F: Father).

After the wild-type and mutant plasmids of FX-WT and FX-Ile265Thr were successfully constructed and respectively transfected into HEK293 cells, the FX antigen levels in the culture supernatant and cell lysate of the transfected cells were detected by ELISA. The results(Figure 3A) showed that the FX: Ag of the mutant in cell lysates and supernatants were 96.27% (0.963 ± 0.089 ng/mL vs. 1.000 ± 0.145 ng/mL) and 97.98% (1.291 ± 0.106 ng/mL vs. 1.317 ± 0.146 ng/mL) of that of the wild type, respectively, which indicates that FX synthesis and secretion are normal. Western blot was used to detect the protein in the cell lysates and culture supernatants, as well as plasmas from the proband and his parents. The results did not show obvious difference in the molecular weight (Figure 3B). Together it suggests that the missense mutation Ile265Thr is unlikely to affect the synthesis or secretion of FX.

### 3.4. Activation of Recombinant FX-WT and FX-Ile265Thr

**Figure 4:**
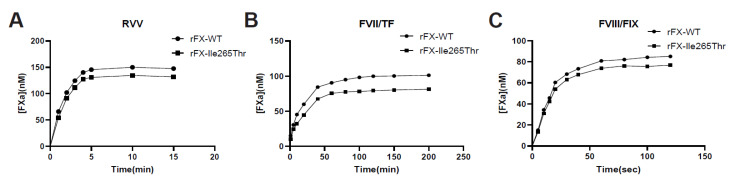
(**A**). Activation of recombinant FX-WT and FX- Ile265Thr by RVV. RVV(100 nM) was used to activate 150 nM FX-WT (●) or FX-Ile265Thr (▇) at 37 °C. The concentration of FXa was monitored by measuring the rate of chromogenic substrate hydrolysis as described under “Materials and Methods.” (**B**). Activation of recombinant FX-WT and FX- Ile265Thr by extrinsic way. 150 nM FX-WT (●) or FX-Ile265Thr (▇) were activated by 60 pM FVIIa in the presence of 500 pM lipidated tissue factor. (**C**). Activation of recombinant FX-WT and FX- Ile265Thr by intrinsic way. 150 nM FX-WT (●) or FX-Ile265Thr (▇) were activated by 5 nM FIXa in the presence of 4 units/mL FVIIIa on 20μM PE:PS:PC vesicles(5:3:2 *w*/*w*). Data are derived from three independent measurements (SD).

Chromogenic assays indicated that at the condition of 100 nM RVV, both 150 nM FX-WT and FX-Ile265Thr were almost completely activated in 5 min while the latter was activated little less efficiently (final FXa 132 nM vs.148 nM, Figure 4A). FX-WT and FX-Ile265Thr have indistinguishable reaction kinetics with the chromogenic substrate S-2765 (Figure 4A). Activation of FX-WT and FX-Ile265Thr by the intrinsic way (FIXa/FVIII) (Figure 4B) and extrinsic way (FVIIa/TF) also showed no obvious difference except FX-Ile265Thr occurred at about 80% of the FX-WT rate (final FXa 81 nM vs.101 nM, Figure 4C). These results indicated that there is no obvious difference between the two FXs in the three activation pathways.

### 3.5. Clotting Activity of Recombinant FX

**Figure 5:**
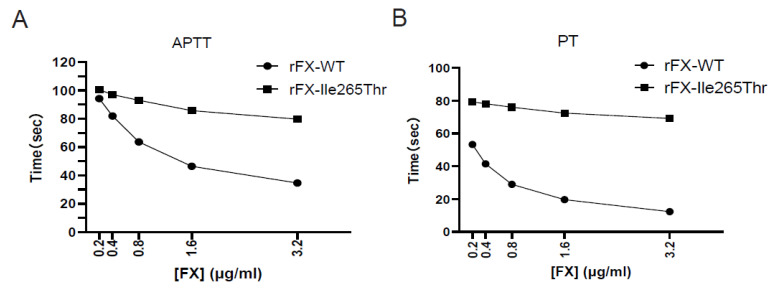
Plasma clotting activities of recombinant FX-WT and FX-Ile265Thr.The clotting activities of FX-WT (●) and FX-Ile265Thr (▇) were determined using FX-deficient plasma by both aPTT (**A**) and PT (**B**) assays as a function of different concentrations(from 0.2 to 3.2 of the zymogens at 37 °C as described in Materials and methods. Data are derived from three independent measurements (SD).

Clotting activities of recombinant FX-WT and FX-Ile265Thr were evaluated using both PT and aPTT assays. In the aPTT assay, the result of the clotting activities indicated that the recombinant FX-WT exhibits a dose-dependent activity and showed activity in normal range when FX-WT > 1.6 μg/mL. However, FX-Ile265Thr only showed slightly increased clotting activity (aPTT shortened 20.7%) when its concentration was rised to 3.2 μg/mL from 0.2 μg/mL) (Figure 5A). In the PT assay, the clotting activity of the recombinant FX-WT exhibits a dose-dependent activity as well. However, FX-Ile265Thr only increased about 12.7% (Figure 5B). These results are in agreement with the clotting data obtained from the patient’s plasma.

### 3.6. Thrombin Generation Assay Analysis

**Figure 6:**
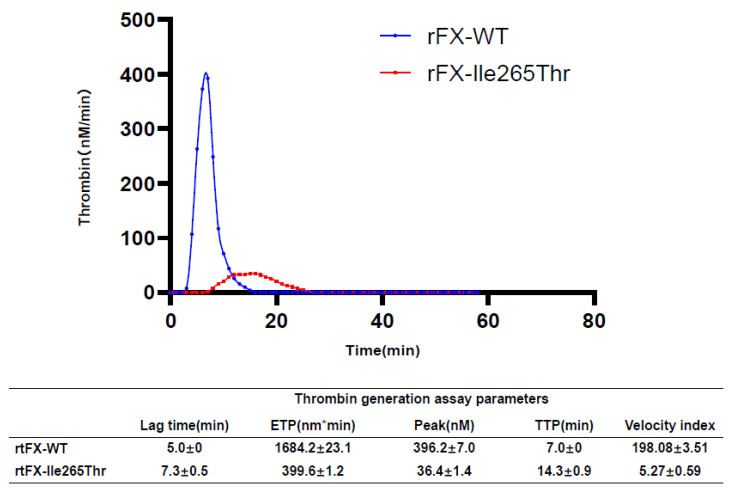
The procoagulant potential of recombinant FX variant. Thrombin generation assays of FX-WT(blue) and FX-Ile265Thr (red) in commercially available FX-deficient plasma using TECHNOTHROMBIN^®^ TGA RC Low kit was measured for 60 min in 1 min intervals at 37 °C. Above is the graph of thrombin generation and below are the parameters of this assay. Data are derived from three independent measurements (SD).

According to the TGA results (Figure 6), the endogenous thrombin potential (ETP) value in the plasma with recombinant FX-Ile265Thr was 399.6 ± 1.2 nM*min, which is markedly lower compared to that of recombinant FX-WT (1684.2 ± 23.1 nM*min), indicating a decreased amount of thrombin generation. Both the peak (36.4 ± 1.4 nM vs. 396.2 ± 7.0 nM) and velocity-index levels (198.08 ± 3.51 vs. 5.27 ± 0.59) of the plasma with FX-Ile265Thr were much lower than those of the recombinant FX-WT, suggesting a decrease in the maximal concentration and rate of thrombin generation. Consistently, both the TTP (Time to peak) and lag-time values of the FX-WT were prolonged compared to those of the FX-Ile265Thr (14.3 ± 0.9 min vs. 7.0 ± 0 min and 7.3 ± 0.5 min vs. 5.0 ± 0 min, respectively). These results indicated that the proband had a defect in thrombin generation due to the missense mutation of FX.

### 3.7. Minigene Assay

**Figure 7:**
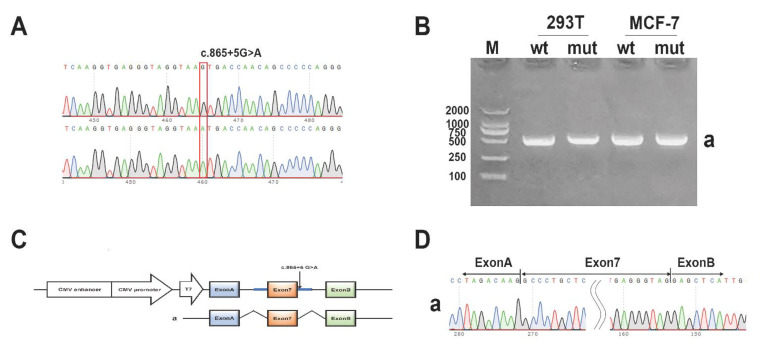
(**A**). Sequencing of the pcMINI-FX-WT and pcMINI-FX-Mut. (**B**). Agarose Gel Electrophoresis for the PCR products in 293T and MCF-7 cells (band a). (**C**)**.** The construction method of the pcMINI-FX-WT/Mut and splicing mode. Modified pcDNA3.1 with double promoters(CMV promoter and T7 promoter) was used (Thermo Fisher Scientific KK) as vectors. The pcMINI-FX-WT/Mut contains exon7 and intron/exon boundaries, as well as exonA and exonB. ExonA and ExonB are sequences with strong splicing recognition used for splicing study before. (**D**). Sequencing of the PCR products using designed primers.

For the splicing mutation IVS7 + 5G > A in F10, the minigene pcMINI-FX-WT/Mut was constructed. The pcMINI-FX-WT/Mut is with intro6(363 bp)-Exon7(118 bp)-intro7(479 bp) inserted into pcMINI vector. Sequencing of the minigenes is showed in Figure 7A. The RT-PCR results showed that in 293T and MCF-7 cells, the wild type had a band of the expected sizes (band a, 507 bp, Figure 7B), and the mutant type had a band of the same size. All the bands were sent for sequencing. Expected splicing mode is shown in Figure 7C. Sequencing results show that band a was a normal splicing band, and its splicing mode is ExonA-Exon7(118 bp)-ExonB. (Figure 7D). According to the minigene assay, the splicing mutation does not affect the normal splicing.

### 3.8. Bioinformatics Analysis

**Figure 8:**
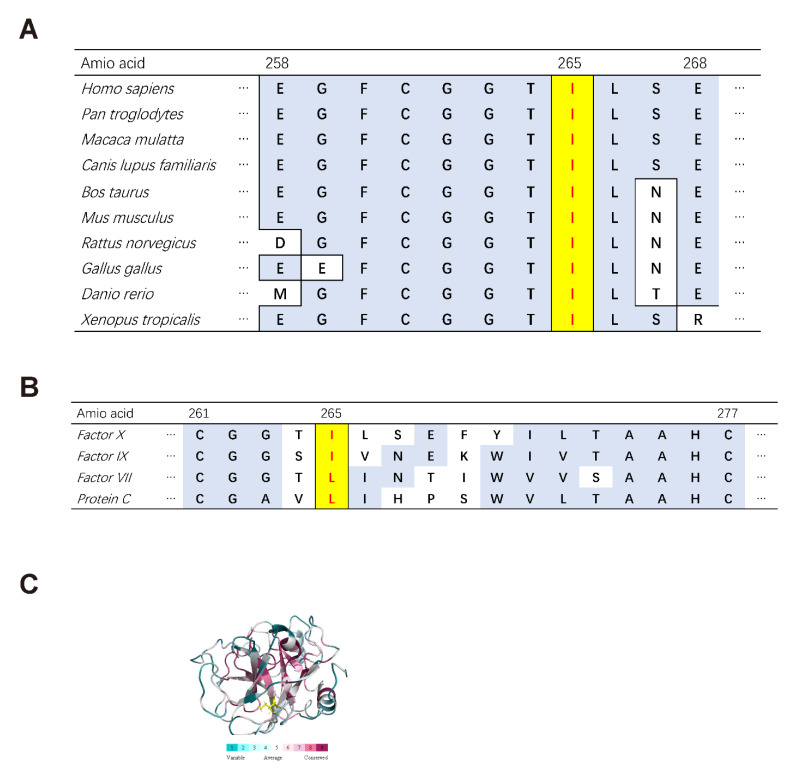
(**A**). Comparison of amino acid alignments around Ile265 among different species. (**B**). Comparison of amino acid alignments around Ile265 among FVII (Factor VII), FIX (Factor IX), PC (Protein C). (**C**)**.** The position of Ile265 (the yellow sticks) in FX and conservation picture of the FX protein (heavy chain) using Pymol.

According to Varcards, the missense mutation c.794T > C: p.Ile265Thr in FX is predicted to be damaging with a damaging score of 0.83 (SIFT: damaging; Polyphen-2: possibly damaging; MutationTaster: disease-causing). Ile265 of FX is a highly conserved amino acid among different species (Figure 8A). It is also high conserved across homologues and is either Ile or Leu in FVII (Factor VII), FIX (Factor IX), PC (Protein C) and even chymotrypsin (Figure 8B). In the FX protein structure, Ile265 is in a region with a highly conservative score (Figure 8C). NNSPLICE analysis of the splicing-site mutation c.865 + 5G > A: IVS7 + 5G > A shows that the original donor site score would decrease from 0.99 to 0.86 and Netgene2 analysis shows that confidence score would decrease from 1.00 to 0.92, which both predict the mutation to affect the normal splicing.

## 4. Discussion

In this article, we analyzed a proband with severe FX deficiency who has experienced abnormal bleeding since childhood. Genetic analysis revealed compound heterozygosity of a missense mutation c.794T > C: p.Ile265Thr and a splicing-site mutation c.865 + 5G > A: IVS7 + 5G > A in the proband. According to the HGMD, the two mutations found in the present Chinese family are novel mutations never described before. According to The Genome Aggregation Database (gnomAD, https://gnomad.broadinstitute.org (accessed on 15 July 2021), a similar variant p.Ile265Ser has been described in one individual but lacking information.

The aPTT and PT of the proband were significantly prolonged to 65.3 s and 80.5 s, respectively. The FX activity of the proband was severely reduced to less than 1% and the FX antigen level was 49.7%. These explains his suffering from abnormal spontaneous bleeding. Genetic analysis of these two mutations in this Chinese pedigree helps confirm congenital FX deficiency and guide clinical practice.

Congenital FX deficiency is generally divided into two types: type I (reduction of both FX activity and antigen) and type II (reduction of FX activity and normal antigen). Here, the proband has reduced FX activity and FX antigen level, however, not proportional. PCCs (Prothrombin complex concentrates), pd-FX/FIX (plasma-derived FX/FIX concentrate), and FFP (Fresh frozen plasma) are the main agents used in the treatment of congenital FX deficiency. However, FFP can be associated with some complications, particularly in children and elderly patients with cardiac disease [25]. PCCs containing FX is associated with the risk of thromboembolic complication due to the high concentrations of FII, FVII, and FIX in these preparations. In addition to FFP and PCC, which were largely used in the past, a recently developed freeze-dried human coagulation FIX/FX concentrate with specified FIX/X content has facilitated prophylaxis. In 2016, a high-purity pd-FX called Coagadex (Bio Products Laboratory, Elstree, UK) was developed as a therapeutic agent for congenital FX deficiency and has since been approved for use in the United States and Europe [10]. While FX replacement with pd-FX is recommended for congenital FX deficiency patients, the use of pd-FX has not yet been approved in China [25,26,27]. In this article, the proband uses PCCs irregularly during daily life while regularly during the perioperative period.

In our research, in-vitro expression study showed normal intracellular synthesis and extracellular secretion of FX-Ile265Thr compared to FX-WT. And activation experiments indicated that in the three pathways, there is no obvious difference between the two FXs except slightly decrease in activation rate of FX-Ile265Thr. Clotting assay and thrombin generation assay both demonstrated low activity of FXa-Ile265Thr in clotting and thrombin generation. All these results suggested that the low activity of the mutant FX-Ile265Thr is caused by a decrease in enzyme activity rather than self-activation. Ile265 of FX is conserved among different species and homologues is either Ile or Leu at this position in FVII (Factor VII), FIX (Factor IX), PC (Protein C), which is in a highly conservative structure. Ile265 is a large and non-polar hydrophobic amino acid present in β-strand C in serine protease subdomain 1 [9], and in a hydrophobic environment between Gly259 to Leu266. The catalytic domain contains the catalytic sites (His236, Asp282, and Ser379), which are essential to the catalytic ability of FX. The replacement by polar neutral amino acid Thr265 may impair the correct folding of this protein or perturb the catalytic triad. Other mutations spatially close to Ile265 may help us understand the possible mechanism. The mutation c.785G > A Gly262Asp was reported by Herrmann et al. [7] and Peyvandi et al. [9] Three patients homozygous for Gly262Asp were all with the FX: C and FX antigen levels both less than 1%. Jayandharan et al. reported a 2-year-old male with FX: C < 1%, who was homozygous for mutation Gly263Arg. At the same position, Gly263Val was reported by Nagaya et al. in a 27-year-old female. Molecular modeling demonstrated that the amino acid substitution at this position disrupts the correct folding of FX. In-vitro expression indicated that Gly263Val protein was secreted less efficiently than the wild-type protein, although they were synthesized normally in the cell [28].

At present, 11 splice-site mutations in the F10 have been reported, but only two have been studied using in vitro splicing and ectopic transcript analysis [13,29]. To study the mutation c.865 + 5G > A: IVS7 + 5G > A, we used in vitro splicing and ectopic transcript analysis. However, due to the low expression of FX in the whole blood, no targeted band was found by agarose gel electrophoresis after 3 rounds of nested PCR amplification (primers are listed in Supplementary Table S1). Minigene assay was used to study in vitro splicing. However, the minigene vectors in two kinds of mammalian cell lines showed no difference between FX/WT and FX/Mut (c.865 + 5G > A: IVS7 + 5G > A). Although the results of the minigene assay showed no difference, the pedigree survey, genetic analysis, and parameters of the proband and his family members all indicated that the mutation c.865 + 5G > A: IVS7 + 5G > A could contribute to causing FX deficiency along with Ile265Thr. NNSPLICE and Netgene2 analysis of the splicing-site mutation c.865 + 5G > A: IVS7 + 5G > A both predict the mutation to affect the normal splicing as well. The splicing reporter minigene assay can nevertheless present some limitations. In the minigene assay, the heterologous cellular system used in the minigene assay may not fully reflect the splicing regulatory process involved in the affected tissue [30]. However, Ding et al. reported a 46-year-old Chinese male, who has factor VII (FVII) deficiency with IVS1 + 5G > A in F7, which resulted in two novel aberrant patterns of splicing [31]. Moreover, IVS2 + 5G > T, IVS3 + 5G > A, IVS7 + 5G > A in F7 all resulted aberrant patterns of splicing [32,33,34]. In F9, IVS1 + 5G > A, IVS1 + 5G > T, IVS1 + 5G > C, IVS2 + 5G > A, IVS2 + 5G > T, IVS2 + 5G > C, IVS3 + 5G > A, IVS3 + 5G > C, IVS4 + 5G > A are all reported previously to be associated with haemophilia B. In PROC(protein C), IVS3 + 5G > A, IVS5 + 5G > A, IVS5 + 5G > T, IVS5 + 5G > C are all reported to result in protein C deficiency [34,35]. Moreover, IVS5 + 5G > A and IVS19 + 5G > A in F8 are demonstrated to be associated with haemophilia A caused by impaired splicing patterns [36,37]. Considering the homology of FX, FVII, FIX, and protein C, the mutation we identified, IVS7 + 5G > A in F10 is quite likely to cause FX deficiency.

In the present study, we observed that the mutation c.794T > C: p.Ile265Thr or c.865 + 5G > A: IVS7 + 5G > A alone could cause decreased FX concentration or activity without causing clinical bleeding. However, when present together, they caused severe bleeding. Therefore, we may preliminarily conclude that these two sequence variant sites have a dose-effect relationship and cause FX deficiency. Similar cases of compound heterozygous mutations have been also reported by many studies [14,38,39,40,41,42]. Our research reported two novel unreported mutations for the first time, and conducted in vitro functional studies on them to find potential pathogenic mechanism also. However, there are some limitations for our research. Firstly, a whole genome sequencing is necessary to rule out potential pathogenic deep intronic variant in the future. Secondly, we will adjust and repeat our minigene assay to simulate a situation closer to the endogenous expression. Nevertheless, how the mutation Ile265Thr impairs the enzyme activity of FX and how the splicing mutation IVS7 + 5G > A of FX functions need further functional research in depth to be done.

## Figures and Tables

**Table 1:** Demographics and coagulation parameters of the proband and family members.

Family Members	Sex	Age (Years)	aPTT (s)	PT (s)	FX Activity %	FX Antigen %
Proband	M	28	65.3	80.5	<1	49.7
Mother	F	56	25.3	12.2	50.3	43.2
Father	M	58	26.4	11.3	57.2	72.0
Sister	F	32	30.0	12.7	40.7	45.3
Wife	F	26	24.3	12.3	93.0	112.2
Son1	M	4	29.8	12.4	47.8	42.3
Son2(twin)	M	1 month	38.2	12.7	-	-
Son3(twin)	M	1 month	37.6	13.1	-	-

aPTT, activated partial thromboplastin time; PT, prothrombin time; Reference interval (*n* = 50): aPTT: 28.0–43.5 s; PT: 11.0–16.0 s; FX (factor X) activity: 70–120%; FX antigen: 80–120%.

## Supplementary Materials

The following are available online at https://www.mdpi.com/article/10.3390/genes12101521/s1, Table S1: PCR primers and sequence in “Materials and methods”.
