# Supplementary material for: Characterization of a Missense Mutation in the Catalytic Domain and a Splicing Mutation of Coagulation Factor X Compound Heterozygous in a Chinese Pedigree
Source: Genes (Basel). 2021 Sep 27;12(10):1521. doi: 10.3390/genes12101521 (PMC8535979; doi:10.3390/genes12101521)
Supplement: Supplementary file 1 [file genes-12-01521-s001.zip › genes-1343620-supplementary.pdf]

Supplementary Table S1 PCR primers and sequence in “Materials and methods”.

Aim	PCR primers	sequence（5’-3’）
For gene analysis of the proband and family members	FX-0F	GATGGTCACGGGAAGGTCAC
	FX-0R	TTTTGGGTCTGAAGGGCGAG
	FX-1F	CTGGAAACTACAAAATGAGCACGA
	FX-1R	CCCAGGGAGCCACAGC
	FX-2F	AGGAAGAAATAATGAGGCGTGTG
	FX-2R	TCGCCGGGAAGGAGTCA
	FX-3F	TGACTGAAGCCACATCTTCACAG
	FX-3R	GTTTTTTGCTTTTACCGAATACACT
	FX-4F	CCATCCTTCCAGTTGGTCCC
	FX-4R	TTTGGGGGACATATTGGGGC
	FX-5F	GCCTGGCTTCTGGCTACAC
	FX-5R	GGGAAGCTGAGGTTAAACAGC
	FX-6F	TCCATTGTTCACAGGCGGTC
	FX-6R	TGGAGCTCACAGCCTCCTT
	FX-7F	TGTGTCGTGTGCATGAGACTT
	FX-7R	GAAGGGCTGGGCTGGTTC
	FX-8F	AACGTGTGAGACAGGACCAG
	FX-8R	ATCTGGGGAAAGGAATGCCC
For patient’s periphery FX ectopic transcript	F10-E2-F	ACGAGGGCCAATTCCTTTCTTG
	F10-E8-1R	CCTCCTGCTTGGTGTCGTAGCC
	F10-E4-F	GCACCTGTTTAGAAGGATTCGAAG
	F10-3UTR-2R	GAGACAAACCAGGCCTTGAGTG
	F10-E6-F	CAACCAGACGCAGCCTGAGAG
	F10-3UTR-3R	GATGCAGGAAGGTATCTGGGGA
For the construction of minigene pcMINI-FX-WT/Mut	23591-F10-F	CTGGAGGGCTCACTGGAGGG
	26022-F10-R	GGACATGCTCTCGCACATCCG
	MINI-F10-KpnI-F	GGTAGGTACCTGTTTCTGCACAGACTGGCGTC
	MINI-F10-BamHI-R	TAGTGGATCCGCATTTGTAGCTTGTGTTGGG
